# Superficial Nœvus

**Published:** 1844-10-19

**Authors:** 


#### SUPERFICIAL NŒVUS.

Mr. Tucker has cured several cases of superficial nœvus (claret stains) by the application of the nitrate of silver in substance. In some of the instances the application of the caustic for a few times was quite sufficient to eradicate the deformity.—*Ibid.*

